# Impact of Relative Energy Deficiency in Sport (REDs) on Bone Health in Elite Athletes: A Retrospective Analysis

**DOI:** 10.1002/jcsm.70082

**Published:** 2025-10-01

**Authors:** Felix N. von Brackel, Robert Munzinger, Mikolaj Bartosik, Alexander Simon, Florian Barvencik, Ralf Oheim, Michael Amling

**Affiliations:** ^1^ Department of Osteology and Biomechanics University Medical Center Hamburg‐Eppendorf Hamburg Germany

**Keywords:** bone stress injuries, catabolic bone metabolism, elite athletes, relative energy deficiency in sport (REDs), stress fractures

## Abstract

**Background:**

Relative energy deficiency in sport (REDs) is associated with impaired performance and compromised bone health in elite athletes. While reduced bone mineral density (BMD) and increased risk for bone stress injuries are well documented, the underlying metabolic mechanisms remain poorly understood. This study investigates the bone metabolism in athletes with REDs and its impact on BMD and bone microstructure.

**Methods:**

We retrospectively analysed data from 82 elite athletes (30.5% females, age 23.4 ± 7.6 years) who presented to our outpatient clinic. The diagnosis of REDs was made according to the International Olympic Committee REDs Clinical Assessment Tool Version 2 (IOC REDs CAT2), and athletes were categorised into strength‐based vs. endurance‐based sports. Laboratory assessment of calcium and bone metabolism included bone turnover markers such as osteocalcin, procollagen type 1 N‐terminal propeptide (P1NP) and urinary deoxypyridinoline (DPD). Areal BMD with corresponding *Z*‐score was measured by dual‐energy X‐ray absorptiometry (DXA) at the lumbar spine and hip. Volumetric BMD and bone microstructure were assessed by high‐resolution peripheral quantitative computed tomography (HR‐pQCT) at the distal radius and tibia.

**Results:**

REDs was diagnosed in 24% of the athletes, and stress fractures were observed more frequently in athletes with REDs compared with those without REDs (70% vs. 25%, *p* < 0.001). Athletes with REDs showed significantly lower haemoglobin and haematocrit levels (*p* < 0.05). Osteocalcin and P1NP were reduced in REDs compared with athletes of strength‐based disciplines (*p* < 0.01), while urinary DPD/creatinine and calcium excretion were elevated (*p* < 0.05), indicating suppressed bone formation and increased bone resorption, respectively. Athletes with REDs exhibited significantly reduced *Z*‐scores at the lumbar spine and hip compared with strength and endurance athletes without REDs (*p* < 0.05). HR‐pQCT revealed significantly lower bone volume to tissue volume and trabecular BMD at the distal radius and tibia, with more pronounced effects at the load‐bearing tibia (*p* < 0.01). Similarly, trabecular number and cortical thickness were reduced in REDs, while no differences were observed in trabecular thickness.

**Conclusion:**

Athletes with REDs are characterised by a catabolic bone metabolism, marked by reduced bone formation alongside increased bone resorption. The resulting metabolic imbalance compromises skeletal adaptations to mechanical loading and contributes to decreased BMD and deteriorated bone microstructure, particularly at weight‐bearing sites. These findings underscore the shared key features of REDs and cachexia and highlight the need for early identification and management of REDs to prevent bone stress injuries and preserve athletic performance. Monitoring bone metabolism may support targeted treatment and improve outcomes in affected athletes.

## 1 Introduction

Although elite athletes and cachectic individuals seem to lie at opposite ends of the physiological spectrum, a specific subset of athletes [those affected by relative energy deficiency in sport (REDs)] may share striking similarities with cachexia in terms of energy imbalance and catabolic tissue remodelling. Despite intense physical training, these athletes often fail to gain adequate muscle and bone mass and may even experience progressive loss of bone quality, increased risk of stress fractures and impaired sport performance.

Elite sport places particular demands on the body and mind of athletes. While athletes often achieve remarkable performance, cases of elite athletes not reaching their potential are frequently observed in clinical practice. These athletes may even experience declining sport performance, often along with complications like fractures. Such cases are often associated with problematic low energy availability (LEA) relative to the body's high demands. Originally recognised as the ‘Female Athlete Triad (FAT)’ in females, this concept has since evolved. In 2014, an IOC consensus statement replaced the term ‘Female Athlete Triad’ by ‘Relative Energy Deficiency in Sport’ (RED‐S [1]), now updated to REDs [2].

REDs is now recognised as a syndrome affecting both genders, though 80% of reported cases involve females [2]. It is defined as ‘a syndrome of impaired physiological [ … ] functioning experienced by [ … ] athletes [ … ] caused by exposure to problematic [ … ] low energy availability. The detrimental outcomes include [ … ] musculoskeletal health [ … ] haematological health, which can all […] lead to […] increased injury risk and decreased sports performance’ [2]. Decreased bone mineral density (BMD) and reduced bone microstructure are documented across disciplines [2, 3, 4, 5, 6, 7, 8, 9, 10, 11, 12, 13, 14], yet bone metabolic data linking this deterioration to specific metabolic conditions remain limited. Some studies note reduced bone metabolism [15, 16, 17], but direct links to BMD and microstructure are lacking. Furthermore, most research focuses on females, while male athletes are also affected. Therefore, while LEA is known to reduce BMD and impair bone microstructure, potentially through metabolic mechanisms, direct metabolic data linking these changes within the same individuals are currently lacking.

We aim to investigate bone metabolism in elite athletes by comparing those with and without REDs and examine the relationship between metabolic status and changes in BMD as well as bone microstructure. We hypothesise that REDs represents a cachexia‐like, catabolic state with reduced bone formation, leading to decreased BMD due to increased bone resorption, while suppressed formation can also lead to reduced bone mass. Additionally, we propose that this reduction in BMD is associated with compromised bone microstructure and three‐dimensional mineralisation. Our goal is to enhance understanding of the mechanisms underlying the well‐documented BMD reduction in REDs and to raise awareness among clinicians and athletes about the detrimental impact of REDs on training success and sports performance through increased injury risk and decreased athlete availability.

## 2 Materials and Methods

### 2.1 Athletes

Patient files from June 2013 to November 2023 from the Department of Osteology and Biomechanics (University Medical Center Hamburg‐Eppendorf, Germany) were screened for the diagnosis of REDs or similar diagnoses such as female/male athlete triad, amenorrhea and oligomenorrhea in elite athletes, as well as for elite athletes without REDs. Our study focuses on biological sex, while remaining mindful of equity and inclusivity principles in the broader context of sports medicine research. The term ‘athletes’ in this study refers to patients who presented to our outpatient clinic and are described as ‘athletes’ for clarity within the manuscript.

### 2.2 Clinical Characteristics

Elite was defined as a minimum of 21 h of sport per week [18] or any athlete that competes at a national or international level [19]. Additionally, the athletes were categorised according to the McKay classification [20]. REDs was defined as a clinical diagnosis based on the 2023 International Olympic Committee consensus statement on REDs [2]. Additionally, female athletes were evaluated for amenorrhoea, while male athletes were assessed for missing morning erections. Fracture frequency and types were extracted from patient files, assessed through structured clinical interviews, and confirmed by images in the outpatients' Picture Archiving and Communication Systems. Interviews included questions about weekly training time, type of sport and current performance outcomes. Additionally, patient‐centred evaluations were conducted to assess changes in performance over time and compare these changes to expected performance. Clinical treatment was assessed by means of vitamin D and calcium substitution as well as (off‐label) bone‐specific drug treatment.

Athletes were retrospectively stratified into REDs severity/risk groups (green, yellow, orange, red) based on the CAT2 classification [21]. This classification reflects the minimum severity level, as not all data were available for categorisation; any additional information would either confirm the assigned category or indicate a more severe classification. Green represents no REDs to very low REDs severity, yellow indicates mild REDs severity, orange corresponds to moderate to high severity and red reflects very high to extreme severity [21].

### 2.3 Biochemical Assessment

Blood and urine sampling was performed as part of the clinical routine to quantify bone metabolism. Haemoglobin (Hb) and haematocrit (Hct) were assessed as clinical markers of haematological health. Bone metabolism was evaluated through serum levels of calcium and phosphorus, as well as bone formation markers including osteocalcin (BGLAP), bone‐specific alkaline phosphatase (bAP) and procollagen type I N‐terminal propeptide (P1NP). Serum parathyroid hormone (PTH) levels were measured to assess calcium regulation. Urine samples were analysed to quantify bone resorption using deoxypyridinoline (DPD) relative to creatinine (DPD/crea) and to determine urinary calcium excretion per urinary creatinine. All blood and urine samples were analysed at the Institute of Clinical Chemistry and Laboratory Medicine, University Medical Center Hamburg‐Eppendorf, Germany.

### 2.4 DXA Areal Bone Mineral Density (aBMD)

Bone densitometry was conducted during routine clinical visits using a Lunar iDXA (GE Healthcare, WI, USA), following ISCD guidelines [22] to quantify BMD. Measurements were taken at the lumbar spine (L1 to L4) and both hips, including the femoral neck and total hip. For each patient, *Z*‐scores were calculated using the manufacturer's NHANES reference values. The lowest *Z*‐score from the right and left total hip was selected, and the mean *Z*‐score for L1–L4 was used for the lumbar spine.

### 2.5 High‐Resolution Peripheral Quantitative Computed Tomography (HR‐pQCT) Measurements

Due to the extended study period (2013 to 2023), high‐resolution peripheral quantitative computed tomography (HR‐pQCT) was performed using either a first‐ or a second‐generation device (XtremeCT or XtremeCT II, Scanco Medical AG, Brüttisellen, Switzerland). The first‐generation device has an isometric voxel size of 82 μm; the second‐generation device has a isometric voxel size of 60.7 μm. Scanning followed established guidelines using a fixed offset distance (9.5 mm for radius and 22.5 mm for tibia in the first‐generation device and 9 and 22.0 mm in the second‐generation device respectively; the edge of the radiocarpal joint surface and the tibial plafond were used as landmarks) [23]. Image segmentation and evaluations were done according to the manufacturer's established routines and scripts.

Results from the first‐generation device were recalculated according to Manske et al. [24] to match the second‐generation. Bone volume to tissue volume (BV/TV), trabecular volumetric bone mineral density (Tb.vBMD), total volumetric BMD (Tt.vBMD), trabecular number (Tb.N), trabecular thickness (Tb.Th) and cortical thickness (Ct.Th) were considered. Scans with motion artefacts were excluded [23, 25]. While in the second‐generation device all parameters are measured directly, some of the first‐generation parameters (e.g., BV/TV, Tb. Th, Ct.Th) are derived parameters [23]. Measurements are presented as a percentage of the median for each specific age and sex group. Hansen et al. were used for first‐generation reference values and Whittier et al. for second‐generation values [26, 27].

### 2.6 Statistical Evaluation

Normally distributed data were analysed by ANOVA with post hoc Šidák correction for multiple comparisons. For non‐normally distributed data, a Kruskal–Wallis test was used with Dunn's multiple comparison test. Frequency comparisons were performed using the chi‐square test. Tests and graphs were performed using the GraphPad Prism 9 (version 9.0.2, GraphPad Software LLC, California, USA). Confidence intervals (CI) are referred to as 95% CI. If no exact means and confidence intervals (CI) were available, we reported the 95% CI_parameter_ of the difference and the mean difference (MD_parameter_) for ANOVA and the mean rank difference (MRD_parameter_) for Kruskal–Wallis tests with Dunn's multiple comparisons.

## 3 Results

### 3.1 Athletes

**FIGURE 1:**
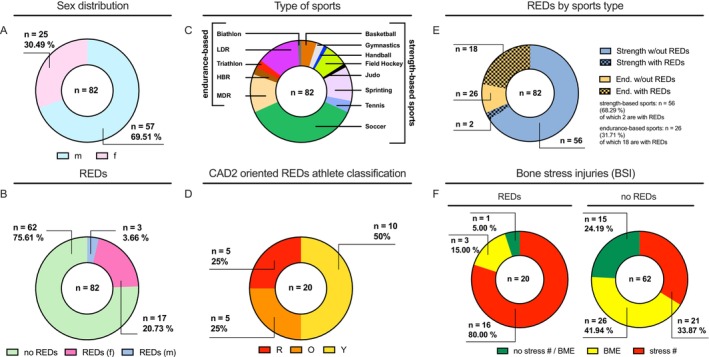
Descriptives of the study cohorts: The majority (69.51%) of the 82 athletes were male (A). Of these athletes, 20 (24.39%) had clinically diagnosed REDs (relative energy deficiency in sports) as shown separately for males and females in panel (B). There was a wide distribution of sports, which were categorised into strength and endurance sports to account for the different known bone density manifestations (C). The CAD2 oriented classification of the REDs athletes revealed that 25% of the athletes were at very high severity (red), 25% at moderate to high severity (orange) and the remaining 50% at mild severity (yellow) (D). Significantly more (*p* < 0.0001; 18 of 26) endurance athletes had clinically diagnosed REDs, whereas only two of 58 strength athletes had REDs (E). Among the elite athletes with REDs, significantly more athletes (*p* < 0.0014; 80%) had a stress fracture, while among the other athletes without REDs, only 33.87% had a stress fracture. BME, bone marrow oedema; f, female; HBR, horseback riding; LDR, long‐distance running; m, male; MDR, middle distance running; REDs = relative energy deficiency in sport; stress #, stress fracture; w/out, without.

Eighty‐two athletes were identified. Twenty‐five athletes (30.49%) were female, and 57 (69.51%) were male (Figure 1A). Fifteen percent of the REDs athletes were male. The age distribution was 29 athletes under 20 years, 39 athletes between 21 and 30, 11 athletes between 31 and 40 and three athletes between 41 and 50. The average age was 23.38 ± 7.57 years, CI [21.52 to 24.90]. According to the McKay classification, all athletes were classified as at least Tier 3, with 51% categorised into Tier 4 or 5.

### 3.2 Clinical Characteristics

Twenty athletes were clinically diagnosed with REDs in accordance with the IOC consensus statement 2023 (Figure 1B) [2]. Among all 82 athletes, 56 were involved in strength‐based disciplines and 26 were endurance athletes (Figure 1C). Of athletes with REDs, 25% were categorised at very high to extreme severity (red), 25% at moderate to high severity (orange) and 50% at mild severity (yellow) using all available data according to the Clinical Assessment Tool Version 2 (IOC REDs CAT2; Figure 1D) [21]. We did not use *Z*‐scores or bone stress injuries as isolated indicators; additional clinical findings were required for group classification to avoid potential bias. REDs was found significantly more often in endurance athletes (18 of the 26, 69.2%) compared with two of the 56 strength athletes (3.6%, *p* < 0.0001; Figure 1E), odds ratio (OR): 60:75. Athletes with REDs had significantly more stress fractures compared with those without REDs (Figure 1F; *p* < 0.0014).

### 3.3 Metabolic Assessment

**FIGURE 2:**
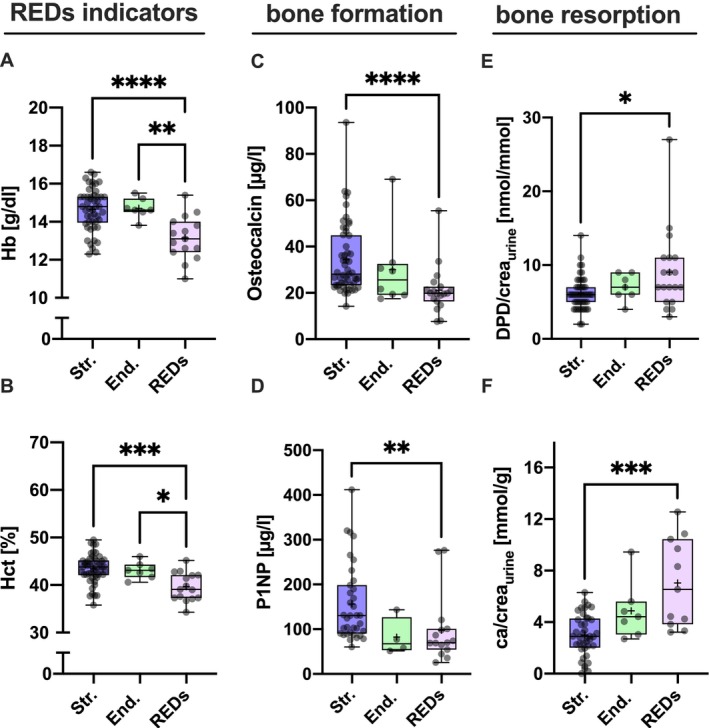
Bone metabolic, laboratory assessment: While elite athletes rely on a good oxygen supply via haemoglobin, elite athletes with REDs showed significantly reduced values of haemoglobin (Hb) and haematocrit (Hct) compared with strength athletes and endurance athletes (*p* < 0.0001). (A, B) Surrogates for osteoblast activity and bone formation such as osteocalcin (C) and procollagen type 1 amino‐terminal propeptide (P1NP, D) were significantly reduced in elite athletes with REDs compared with strength athletes. In contrast, increased bone resorption measured by DPD/crea (E) induces increased calcium excretion (F) in the absence of bone formation, as the released calcium cannot be incorporated into the bone substance. *p < 0.05; **p < 0.01; ***p < 0.001; ****p < 0.0001. ca/crea, calcium in urine per creatinine in urine; DPD/crea, deoxypyridinoline in urine per creatinine in urine; End., endurance based elite athletes; Hb, haemoglobin; Hct, haematocrit; P1NP, procollagen type 1 amino‐terminal propeptide; REDs, athletes with relative energy deficiency in sport; Str., strength‐based elite athletes.

Haemoglobin was significantly lower in elite athletes with REDs compared with strength and endurance athletes without REDs (Figure 2A; Str.:14.64 g/dL ± 1.11 g/dL, CI: 14.33 to 14.96; End.: 14.70 g/dL ± 0.54 g/dL, CI: 14.20 to 15.20; REDs: 13.15 g/dL ± 1.15 g/dL, CI: 12.52 to 13.79; *p* < 0.0001 for REDs vs. Str. and *p* < 0.008 for REDs vs. End.). A similar pattern was seen for haematocrit with significantly lower values for the REDs compared with strength and endurance (Figure 2B; Str.: 43.42% ± 3.03%, CI: 42.55 to 44.29; End.: 43.10% ± 1.75%, CI: 42.55 to 44.29; REDs: 39.64% ± 3.00%, CI: 37.98 to 41.30; *p* < 0.0001 for REDs vs. Str. and *p* = 0.036 for REDs vs. End.). No differences in vitamin D serum levels were detected between groups (Str.: 35.87 μg/L ± 13.79 μg/L, CI: 32.10 to 39.63; End.:34.57 μg/L ± 7.85 μg/L, CI: 27.31 to 41.83; REDs: 50.77 μg/L ± 28.97 μg/L, CI: 36.80 to 64.73; *p* > 0.05). Osteocalcin as a marker for osteoblast activity and bone turnover was significantly lower in REDs compared with strength athletes (Figure 2C; Str.: 34.31 μg/L ± 15.20 μg/L, CI: 30.12 to 38.50; End.: 29.99 μg/L ± 16.88 μg/L, CI: 15.87 to 44.10; REDs: 21.14 μg/L ± 10.29 μg/L, CI: 16.18 to 26.09; *p* < 0.0001 for REDs vs. Str.). A similar pattern was calculated for P1NP as another bone formation marker (Figure 2D; Str.: 155.70 μg/L ± 85.75 μg/L, CI: 126.2 to 185.1; End.: 82.58 μg/L ± 42.01 μg/L, CI: 15.72 to159.4; REDs: 97.63 μg/L ± 76.24 μg/L, CI: 55.41 to 139.8; *p* = 0.0034 for REDs vs. Str.). Furthermore, for REDs, a significantly higher level of bone resorption was detected by means of DPD/crea (Figure 2E; Str.:6.18 nmol/mmol ± 2.21 nmol/mmol, CI: 5.56 to 5.81; End.: 7.00 nmol/mmol ± 1.83 nmol/mmol, CI: 5.31 to 8.69; REDs: 9.05 nmol/mmol ± 6.45 nmol/mmol, CI: 6.43 to 11.68; *p* < 0.05). For calcium excretion, the REDs group had a significantly higher calcium loss than others (Figure 2F; Str.: 2.60 mmol/g ± 1.61 mmol/g, CI: 2.48 to 3.51; End.: 4.87 mmol/g ± 2.25 mmol/g, CI: 2.79 to 6.95; REDs: 7.04 mmol/g ± 3.44 mmol/g, CI: 4.73 to 9.36; *p* < 0.0005 for REDs vs. Str.). Data points available: haemoglobin and haematocrit: 70, osteocalcin: 80, DPD: 79, Ca/Krea: 58 and P1NP: 60.

### 3.4 Densitometry

**FIGURE 3:**
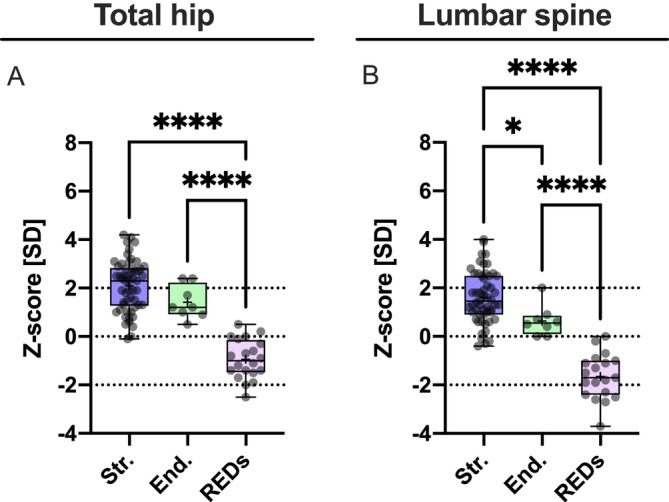
Areal bone mineral density (aBMD): Elite athletes with REDs had significantly lower total hip BMD than others. There was no difference between endurance and strength athletes. (A) When measuring BMD at the spine, there was a significantly lower BMD in athletes with REDs compared with the other groups (B). At the same time, there was also a relatively lower density (but within the reference range) in endurance athletes compared with strength athletes, which could be interpreted as mechanoadaptation to different types of sports. It is noteworthy that six out of 19 (one datapoint missing) athletes with REDs are below the reference range of standardised BMD in the spine and can therefore be considered as having reduced BMD compared with a healthy control group according to the ISCD (International Society of Clinical Densitometry). (B) *p < 0.05, ****p < 0.0001. End., endurance based elite athletes; REDs, athletes with relative energy deficiency in sports; SD, standard deviation; Str., strength‐based elite athletes.

The lowest *Z*‐score across both hips was significantly lower in the REDs group compared with the other groups (Figure 3A; Str.: 2.10 ± 1.06, CI: 1.81 to 2.39; End.: 1.41 ± 0.70, CI: 0.83 to 1.99; REDs: −0.94 ± 0.81, CI: −1.32 to −0.56; *p* < 0.0001 for both, REDs vs. Str. and REDs vs. End.). At the lumbar spine, the mean *Z*‐score was highest in the strength group, lower in the endurance group and lowest in the REDs group (Figure 3B; Str.: 1.60 ± 1.07, CI: 1.31 to 1.89; End.: 0.64 ± 0.63, CI: 0.11 to 1.17; REDs: −1.65 ± 0.93, CI: −2.10 to −1.20; *p* < 0.0001 for REDs vs. Str. and REDs vs. End. and *p* = 0.0391 for End. vs. Str.). 31.58% (6/19) of the REDs elite athletes exhibited a *Z*‐score below the age‐adequate variance (*Z*‐score below −2.0) in the spine and 5.26% (1/19) in the hip. Comparing the subgroups of the REDs athletes based on their REDs CAT2 levels, no differences were seen between the subgroups for aBMD (*p* ≫ 0.05). Two data points are missing.

### 3.5 HR‐pQCT

**FIGURE 4:**
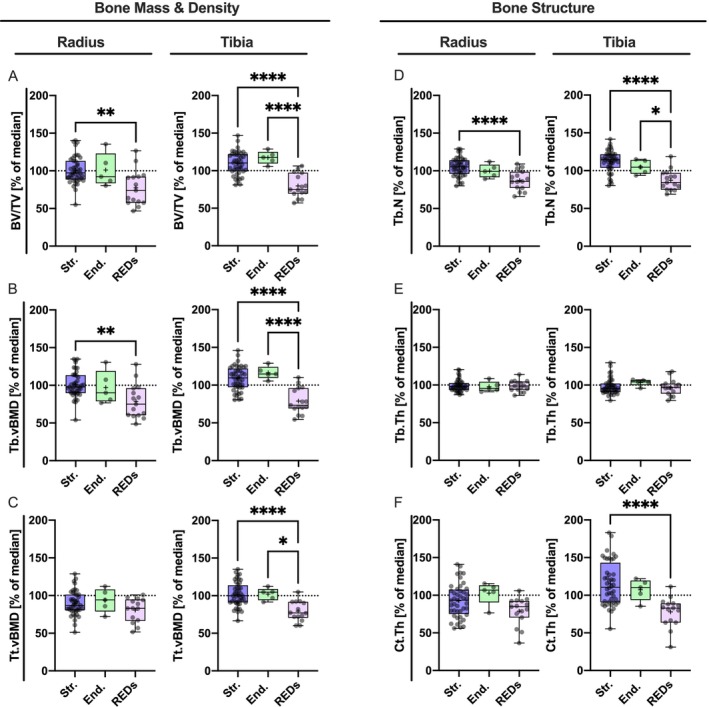
HR‐pQCT measures of bone mass, density and microstructure: REDs athletes exhibited reduced parameters for bone mass, density and microstructure compared with age and sex matched reference median values (A–F). Reduced trabecular bone mass of the REDs group was seen in both the radius and tibia (A) compared with the strength groups in the radius and strength and endurance in the tibia (A). In addition, there was a reduction in Tb.vBMD and Tt.vBMD in the radius in the REDs group compared with the strength athletes (B, C). This difference can also be seen in the tibia, as well as a significant difference compared with endurance athletes (B, C). The analysis of the bone microarchitecture of the radius showed significantly lower measures for the Tb. N in the REDs group (D) but no differences in Tb. Th (E) and Ct. Th (F). In the tibia, Tb. N and Ct. Th were significantly lower in REDs compared with both the strength and endurance groups (H, L). No differences were seen in Tb. Th comparing the three groups (E, F). *p < 0.05; **p < 0.01; ***p < 0.001; ****p < 0.0001. BV/TV, bone volume to tissue volume; Ct.Th, cortical thickness; End., endurance based elite athletes; mgHA/cm^3^, mg hydroxyapatite per cubic centimetre; REDs, athletes with relative energy deficiency in sport; Tb.vBMD, trabecular volumetric bone mineral density; Str., strength‐based elite athletes; Tb.N, trabecular number; Tb.Th, trabecular thickness; Tt.vBMD, total volumetric bone mineral density.

No significant differences in BV/TV, Tb.vBMD or Tt.vBMD between strength‐ and endurance‐based athletes without REDs were seen (Figure 4A–F). At the radius (non‐weight‐bearing), BV/TV and Tb.vBMD were significantly lower in the REDs group compared with strength‐based athletes (BV/TV: *p* = 0.0038, Str.: 100.8 ± 17.85, CI: 95.32 to 106.20; End.: 101.00 ± 22.25, CI: 73.42 to 128.70; REDs: 77.52 ± 23.51, CI: 64.49 to 90.54; Tb.BMD: *p* = 0.0024, Str.: 100.80 ± 16.97, CI: 95.66 to 106.00]; End.: 97.15 ± 22.10, CI: 69.70 to 124.60; REDs: 78.79 ± 22.73, CI: 66.20 to 91.37; Figure 4A,C,D). At the tibia, the REDs group showed significantly lower BV/TV, Tb.vBMD and Tt.vBMD compared with both strength‐ and endurance‐based athletes (Figure 4B,D,F).

For the bone microstructure assessment at the radius, significantly lower Tb. N was observed in the REDs group compared with the strength group (*p* < 0.0001) but no difference between endurance‐based athletes and the REDs group (Figure 4G,I). At the tibia, athletes with REDs were significantly lower in Tb.N compared with strength‐ and endurance‐based athletes, *p* < 0.0001 and *p* < 0.05 respectively (Str.: 112.30 ± 14.19, CI: 108.00 to 116.60; End.: 104.90 ± 9.25, CI: 93.41 to 116.40; REDs: 87.31 ± 13.43, CI: 79.88 to 94.75). Tb.Th was similar for all groups. Ct.Th was significantly lower in the REDs group compared with strength athletes (*p* < 0.0001, Str.: 115.20 ± 29.16, CI: 98.86 to 122.50; End.: 107.20 ± 14.31, CI: 85.27 to 121.80; REDs: 77.95 ± 19.78, CI: 63.73 to 89.14) (Figure 4K,L). No significant differences in HR‐pQCT parameters were observed between REDs subgroups when stratified by REDs CAT2 severity levels (*p* ≫ 0.05). Sixty‐five data points were available.

### 3.6 Therapy

Among all athletes, five athletes with REDs and 15 without REDs were treated with bone‐specific medication. Athletes with REDs were mainly treated with an off‐label osteoanabolic drug (3/5, 60.0%), while athletes without REDs were mainly treated with an off‐label antiresorptive drug (14/15, 93.3%, *p* < 0.01). All athletes supplemented vitamin D.

## 4 Discussion

This is the first study in athletes that provides evidence that diminished bone mass and microstructure in athletes with REDs are associated with a catabolic bone metabolism, which compromises the ability of the bone to remodel and adapt to mechanical loading, particularly at the weight‐bearing skeletal sites. The altered bone metabolism in athletes with REDs, characterised by decreased bone formation and increased bone resorption, reduces the mechano‐adaptive capacity of the skeleton. Consequently, the catabolic bone metabolism, predominantly observed in endurance athletes, contributes to a higher incidence of stress fractures.

We examined 82 elite athletes; a quarter of them were diagnosed with REDs [2]. All athletes with REDs were categorised with at least mild severity according to CAT2, a validated approach of grading REDs severity levels [28]. Given not all information was available for categorisation, the severity may be higher than expected. We have segregated strength and endurance athletes from athletes with REDs because a particularly pronounced bone mass increase is known for strength athletes [29], which is reflected by a significant difference between strength and endurance athletes in the BMD of the lumbar spine (Figure 3B). While non‐REDs athletes mainly experience bone marrow oedema, REDs exacerbate skeletal problems, leading to more severe issues, including a significantly higher incidence of stress fractures.

### 4.1 REDs Induces a Catabolic Metabolism

REDs is a known cause of reduced bone mass and microstructure [2, 6, 7, 12, 30]. However, information about the underlying metabolic mechanisms is scarce [7]. Our results indicate a catabolic bone metabolism with low bone formation and increased bone resorption, leading to calcium loss, particularly detrimental to bone health, in line with recent data from mouse experiments [31]. Interestingly, the REDs‐induced catabolic state shares similarities with cachexia, particularly elevated bone resorption at systemic energy imbalance [32], but may be even more pronounced metabolically, as REDs is also characterised by reduced bone formation in the context of high athletic demands, as reflected by decreased osteocalcin as a bone turnover marker. Both conditions result in impaired tissue remodelling and structural fragility, despite different underlying causes. Training and competition cause microdamage to bone tissue, which is physiologically resorbed and replaced by new bone to preserve mechanical integrity. However, in REDs, while damaged bone is resorbed (both to repair microdamage and to supply calcium in the context of low dietary intake), bone formation is impaired due to reduced osteoblast activity. This suppression of bone formation likely results from insufficient energy availability and inadequate nutritional support for osteoblasts. Accordingly, bone tissue cannot adapt to the high mechanical impact from athletic activity, as the need for increased bone mass through bone modelling is not met due to reduced osteoblast activity. This hinders the body's ability to withstand high loads, leading to the accumulation of microdamage, increased bone resorption, and consequently a loss in bone mass. Thus, REDs creates a catabolic state detrimental to athletes, who need an anabolic state to adapt to high performance stresses. The anabolic state is evident in strength athletes, marked by high bone formation and low resorption. Endurance athletes in our data show an intermediate metabolic state, with no significant differences due to the sample size.

### 4.2 Reduced Bone Mineral Density

BMD is significantly lower in athletes with REDs compared with both strength and endurance athletes without REDs, reflecting the consequences of catabolic bone metabolism. The low bone formation and high resorption aligns with consistent reports of BMD loss in REDs athletes [2, 6, 12, 30] indicating that, unlike healthy athletes, the body cannot adapt to high mechanical loads by increasing bone mass in REDs [6, 7, 33]. Notably, while most athletes with REDs had total hip *Z*‐scores within the normal range, many fell into the reduced BMD range for lumbar spine measurements [34]. This highlights the compartment‐specific adaptations. Most athletes with REDs are endurance athletes, who primarily strain the legs and induce bone mass retention there, while strength athletes typically have high lumbar spine BMD [5]. The spine, with its high trabecular bone volume and large trabecular surface area, is the primary site where changes in bone metabolism manifest. In endurance sports, the spine may experiences less mechanical stress than in strength, reducing the need for bone mass retention. This is reflected in the significantly lower *Z*‐scores of non‐REDs endurance athletes compared with strength athletes, who experience higher mechanical loads on the spine, leading to increased bone mass as noted in the literature [5, 29].

### 4.3 Reduced Bone Microstructure

The reduced areal BMD measured by DXA in athletes with REDs is caused by significant reductions in trabecular and cortical bone microstructure, as well as volumetric BMD measured by HR‐pQCT, consistent with other studies [6, 7, 8]. Interestingly, the differences in athletes with REDs were more pronounced in the tibia than in the radius. Overall, REDs athletes showed a reduced three‐dimensional bone microstructure and density in HR‐pQCT compared with a reference group (Figure 4), indicating the generally reduced metabolic status and impaired bone microstructure and density in athletes with REDs. At the radius, differences were primarily detected between strength athletes and those with REDs. This may be because endurance sports primarily load the legs in healthy athletes, resulting in a higher mechanical stimulus for bone retention and formation at the tibia but not at the radius[5]. Notably, trabecular thickness did not differ among the groups, whereas Tb.N and consequently BV/TV were lower in REDs athletes compared with both in the tibia and to strength athletes in the radius. Furthermore, when comparing strength and endurance athletes, both groups exhibited similar bone microstructure, with a trend toward above‐average values relative to their respective reference groups. Taken together, HR‐pQCT may be, as suggested by the 2023 International Olympic Committee's (IOC) consensus statement, a potential tool to further elaborate the bone mass losses with respect to compartment‐specific affections [2] and is able to decipher the underlying changes of a reduced areal BMD. Furthermore, HR‐pQCT proves to be valuable not only in research settings but may also serve in the early detection of REDs and potentially for monitoring treatment response. When comparing subgroups of the REDs athletes based on their REDs CAT2 levels, no differences between the subgroups have been detected for both aBMD and parameters from HR‐pQCT (data not shown).

### 4.4 Treatment

Treatment of REDs‐associated bone stress fractures is of major clinical importance. While initial management should consider surgical versus nonoperative approaches [13], none of our athletes underwent surgical treatment. Most of the non‐REDs‐associated bone stress injuries of our cohort were treated with an off‐label antiresorptive treatment [35] with a single denosumab (60 mg s.c.) injection [36]. However, in the case of REDs, a significantly higher portion of athletes were treated with an off‐label anabolic treatment to address the particular low osteoblast function seen in the laboratory assessment.

### 4.5 Limitations

While we present a large group of elite athletes characterised for BMD, three‐dimensional microstructure and bone metabolism, this study also has limitations, such as its retrospective nature. In addition, our cohort encompasses more males than females, which may have led to a sex‐based bias; however, it also pinpoints the accuracy of the term REDs instead of Female Athlete Triad given 15% of the REDs athletes to be male. Furthermore, our results are not longitudinal, which is why we cannot quantify the treatment success dependent on the chosen therapy for athletes with REDs. This study did not include athletes from aesthetic or weight‐sensitive sports, which may limit generalisability. Additionally, the observed changes in bone microstructure cannot be attributed solely to REDs, as they may also be influenced by training status, sport type or genetic factors.

## 5 Conclusion and Clinical Implications

Our findings demonstrate that athletes affected by REDs exhibit an imbalance in skeletal metabolism, marked by reduced osteoblast activity and therefore decreased bone formation, alongside increased bone resorption. These metabolic alterations may compromise mechanical adaptation and result in lower BMD and impaired bone microstructure compared with other athletes and healthy controls. Our study underscores the importance for physicians, athletes and coaches to prevent and manage low energy availability and to monitor bone health in athletes at risk in order to reduce the risk of bone stress injuries.

## Ethics Statement

The study was conducted in accordance with local laws and institutional ethical guidelines, as well as the Declaration of Helsinki. Patients (Athletes) provided informed consent for the retrospective analysis of their clinical data in an anonymised manner. Patients in the study were seen as part of the ethics‐committee approved protocol 2022‐100817‐BO‐ff (Ärztekammer Hamburg, Germany).

## Conflicts of Interest

FvB, RM, MB, AS and MA report no competing interests. FB has received consulting fees from Alexion and UCB, speakers' fees from Alexion, UCB, Diasorin and FOMF; support for meeting attendances from UCB and has received institutional research grants from UCB and Alexion. RO has received consulting fees from Kyowa Kirin, Inozyme, Mereo, Ipsen, Pharmacosmos and UCB; speakers' fees from Kyowa Kirin, Inozyme and UCB and has received institutional research grants from Kyowa Kirin, UCB and Inozyme.

## Data Availability

The data underlying this article will be shared upon reasonable request in compliance with national data protection regulations.
